# Urological Content Accuracy as an Unmeasured Dimension in Generative AI Chatbot Communication About Sexual Health After Prostate Cancer

**DOI:** 10.2196/108419

**Published:** 2026-09-15

**Authors:** Bilgi Isler, Ahmet Murat Bayraktar

**Affiliations:** 1 Department of Urology, Konya City Hospital, University of Health Sciences, İstiklal, Adana Çevre Yolu Cd. No:135/1Konya, 42020, Türkiye, 90 5056834407

**Keywords:** auto-netnography, netnography, prostate cancer, sexual minority men, generative AI chatbots

Christiansen and colleagues [1] make a welcome contribution by shifting attention from correctness to interactional framing in a group whose informational needs remain underaddressed. The authors state clearly that they did not set out to assess clinical effectiveness or safety, and they caution in their limitations that the absence of obvious fabrication should not be read as evidence of accuracy. Reading the published outputs from a urological perspective, we offer three observations that may refine future work.

First, the initial prompt states that both radiotherapy and surgery would likely include hormonal therapy. This is a patient utterance, not a claim by the authors, and patients do hold such beliefs. Androgen deprivation is standard in combination with radiotherapy for intermediate- and high-risk disease, but neoadjuvant androgen deprivation around radical prostatectomy is not recommended [2]. What is notable is the response: none of the four systems queried or corrected it, and all four built counseling based on it. Silent acceptance of a premise that is not guideline-concordant is, in our view, among the most consequential failure modes in patient-facing use, and it falls outside the pragmatic accuracy definition used here.

Second, the two treatment arms differ categorically for receptive anal intercourse: after prostatectomy, the gland is absent and prostate-mediated pleasure is permanently lost [3], whereas after radiotherapy, the gland remains in situ and rectal toxicity becomes the limiting factor. Comparative data bear this out: climacturia is far commoner after prostatectomy and anodyspareunia after radiotherapy [4]. Only one output made this explicit. Another described treatment as affecting the sensitivity and function of the prostate, which does not apply once the gland has been removed. The passage quoted twice as an exemplar of compassionate precision proposes a 4‐ to 6-week interval for resuming receptive anal intercourse without reference to anodyspareunia, mucosal fragility, or proctopathy after irradiation.

Third, the statement that no hallucinated content was identified sits alongside a reported support group link misattributed to a Swedish service. We would classify that as a contextual hallucination rather than as a separate category, and the platform responsible is not named. A further unflagged example appears in Multimedia Appendix 2 in Christiansen et al [1], where a hereditary cancer organization is presented as a resource for LGBTQ+ (lesbian, gay, bisexual, transgender, queer) individuals with cancer.

These observations do not undermine the authors’ interpretive aim but indicate where a complementary design would add value. We suggest three additions: (1) a prespecified urology-anchored content checklist scored independently by two clinicians, covering the prostatectomy versus radiotherapy distinction, anodyspareunia and rectal toxicity, climacturia, penile length change, and the expected duration and reversibility of androgen deprivation; (2) deliberate inclusion of premises that are not guideline-concordant, with correction rate as an explicit end point; and (3) per-model reporting of every identified error, with exact query dates, session conditions, and repeated generations, as the CHART (Chatbot Health Advice Reporting Tool) statement the authors followed recommends [5].

Communicative warmth and clinical adequacy are separable qualities. This study measures the first with care, and we would welcome a companion study that measures the second.

## Abbreviations

CHART: Chatbot Health Advice Reporting Tool
LGBTQ+: lesbian, gay, bisexual, transgender, queer
